# Research Status of the Knowledge-Attitude-Practice Theory Model in Gastric Cancer Prevention

**DOI:** 10.7759/cureus.64960

**Published:** 2024-07-19

**Authors:** Kai Kang, Mary Angelica P Bagaoisan

**Affiliations:** 1 Graduate School, Angeles University Foundation, Angeles City, PHL

**Keywords:** health promotion, health education, kap theory model, gastric cancer prevention, gastric cancer

## Abstract

Gastric cancer, as a malignant tumor with high morbidity and mortality, poses a serious threat to people’s health. The knowledge-attitude-practice (KAP) theory model shows important value in gastric cancer prevention by improving health knowledge, changing health attitudes or beliefs, and guiding healthy practice. At present, the application of this model in gastric cancer prevention has gradually received attention. This article reviews the current application status of the KAP theory model in gastric cancer prevention and analyzes the challenges, countermeasures, and development prospects in order to provide a reference for gastric cancer prevention.

## Introduction and background

Gastric cancer is one of the most common malignant tumors in the world. Its morbidity and mortality rates remain high, posing a serious threat to human health and life [1,2]. According to the latest epidemiological studies, the occurrence of gastric cancer is closely related to multiple factors, including genetic factors, environmental factors, lifestyle, and *Helicobacter pylori *infection [3]. Although modern medicine has made certain progress in the diagnosis and treatment of gastric cancer, the prognosis of gastric cancer patients is still not optimistic [4]. Therefore, it is particularly important to prevent the occurrence of gastric cancer.

The knowledge-attitude-practice (KAP) theory model is a theoretical framework widely used in the fields of health education and behavioral intervention [5]. The theory believes that knowledge is the basis for forming correct attitudes and practice changes, while attitude is the driving force behind practice changes. Ultimately, the purpose of preventing diseases and promoting health is achieved through changes in practice [6].

In the field of gastric cancer prevention, the KAP theory model has important application value. By popularizing and educating people about gastric cancer-related knowledge, they can improve their understanding of gastric cancer risk factors, thereby changing their knowledge of gastric cancer prevention and ultimately prompting them to adopt active and effective prevention practices. However, there are still some problems and challenges in the application of the KAP theory model in gastric cancer prevention [7-9]. Therefore, in-depth research on the application of the KAP theory model in gastric cancer prevention and exploring more effective intervention strategies and methods have important theoretical and practical significance for reducing the incidence of gastric cancer and improving the health level of the population [10,11].

## Review

Concept and development trend of gastric cancer

Gastric cancer, originating from the gastric mucosal epithelium, represents more than 95% of malignant tumors affecting the stomach [3]. Global statistics for 2022 indicate a significant impact, with an estimated 968,000 new cases and approximately 660,000 deaths attributed to gastric cancer worldwide, ranking fifth in both incidence and mortality among all cancers [1]. Looking ahead, projections suggest a troubling trend, with anticipated global cases increasing by 62% to 1.77 million by 2040 [4]. Asia, particularly China, bears a substantial burden of gastric cancer cases. In 2022 alone, China is expected to report around 358,700 new cases, positioning it as one of the highest-incidence regions globally. Among these cases, males are disproportionately affected, comprising 246,600 new diagnoses compared to 112,100 among females. Gastric cancer in China also accounts for significant mortality, with an estimated 260,400 deaths projected for 2022, ranking it fourth in cancer-related deaths within the country [2]. The etiology of gastric cancer involves various carcinogenic factors, many of which are preventable or treatable [3]. Therefore, emphasizing early detection, prompt diagnosis, and timely treatment are crucial strategies in combating this disease.

KAP theoretical model

Concept and Connotation

The KAP theory model was proposed by American Everett M. Rogers in the late 1950s and early 1960s. It is a behavioral intervention theory that can effectively change patients’ bad practices. The KAP theory includes three stages: acquiring knowledge, building attitude, and constructing practice. It is based on knowledge and driven by individual attitudes, ultimately achieving practice change [6]. Knowledge is the foundation and premise for practice change. It refers to the information, understanding, and skills acquired by individuals through learning and experience accumulation. The acquisition and accumulation of knowledge are crucial for individuals to form correct cognitions and attitudes. Attitude is the driving force behind practice change. It is based on the individual’s understanding and internalization of knowledge, forming a stable and lasting psychological state. The individual’s cognition and attitude toward practice ultimately influence the formation and change of practice. Practice is the direct embodiment of knowledge and attitude, and it is also the ultimate goal of practice change. Practice is the actions and reactions taken by individuals in specific environments and situations, and it is directly influenced and dominated by knowledge and attitude. The KAP theoretical model has wide application value in many fields, especially in medical care, health education, and nursing management. Strengthening individuals’ understanding and cognition of disease knowledge promotes their beliefs and attitudes toward treatment, thereby guiding them to adopt correct health practices [5]. In summary, the KAP theoretical model emphasizes the progressive and interdependent relationship between knowledge, attitude, and practice and provides strong theoretical support and practical guidance for individual behavior change.

Application of the KAP Theory Model in the Medical Field

The application of the KAP theoretical model in the medical field not only demonstrates its significant value in improving the quality of medical services and patient health but is also an important innovation in the development process of the medical industry.

In terms of disease health education, Mao et al. [12] conducted continuity nursing for elderly patients with benign prostatic hyperplasia based on the KAP theoretical model. By deeply exploring the patients’ knowledge status, attitude structure, and practice model, they tailored a personalized continuity nursing plan for the patients, thereby ensuring that the patients could fully understand and follow the health guidance. The results showed that the research group exhibited higher treatment compliance (P = 0.002) and greater nursing satisfaction compared to the control group (P = 0.014).

In terms of nursing management, the KAP theoretical model ensures the efficiency, safety, and quality of nursing services by strengthening the professional knowledge, professional attitude, and practice standards of nursing staff. For example, in order to reduce the risk of needlestick and sharps injuries faced by nurses, Yang et al. [13] developed a KAP theoretical model to conduct behavioral interventions on nurses. The results showed that the number of people who reported needlestick and sharps injuries decreased from 42 to 15 within three months after the intervention. On the other hand, in order to improve the ability of hospital infection prevention and control, Zhang et al. [14] used the KAP theoretical model to investigate the current status of infection prevention and control among hospital nursing staff. The results showed that the older the age, the higher the education level, and the higher the professional title, the better the KAP score for hospital infection prevention and control (P < 0.05), suggesting that researchers can use the KAP theoretical model to deeply analyze the relationship between nursing staff’s knowledge, attitude, and practice and provide a more accurate and scientific decision-making basis for nursing management.

In terms of nursing education, Zhou et al. [15] adopted a qualitative research method and used the KAP theoretical model to analyze the deficiencies in the knowledge and practice of nursing students in responding to large-scale public health emergencies such as the COVID-19 pandemic. A total of 12 subject headings and 41 sublevel headings were identified. Based on this analysis, it was found that nursing students had three major problems in responding to large-scale public health emergencies: lack of knowledge in infectious disease epidemiology, insufficient evidence-based practice skills, and insufficient problem-solving practice. The aim was to prepare for the development of targeted capacity-enhancing education for nursing students.

In terms of disease prevention and health care, the KAP theoretical model can achieve the goal of preventing the occurrence of diseases by popularizing disease knowledge, enhancing prevention awareness, strengthening personal protection, and advocating healthy lifestyles, thereby strengthening community, family, and individual health care. Mubin et al. [16] found that most community healthcare providers (90.91%) and health assistants (96.06%) did not receive cancer-related training and proposed to popularize disease knowledge among these people in order to improve disease prevention and healthcare capabilities of primary medical institutions.

Application, challenges, countermeasures, and prospects of the KAP theoretical model in gastric cancer prevention

Application of the KAP Theory Model in Gastric Cancer Prevention

The current application status of the KAP theoretical model in gastric cancer prevention has received increasing attention, and it has shown significant effects in improving public knowledge of gastric cancer, changing bad life practices, and promoting healthy practices. From a horizontal perspective, the KAP theoretical model can be applied to objects centered on gastric cancer patients, such as gastric cancer patients themselves, their families, medical staff, etc. Research results show that gastric cancer patients who received KAP health education intervention had higher positive attitudes, self-stress relief, self-decision-making, and total scores than the control group, and the difference was statistically significant (t = 4.58, 3.02, 12.87, and 4.11, respectively; all P < 0.01) [17]. From a longitudinal perspective, the KAP theoretical model can be applied throughout the entire process of gastric cancer development, and it can be used for intervention in all stages of the tertiary prevention of gastric cancer. Regarding the primary prevention stage of gastric cancer, Malek et al. [7] developed a questionnaire based on the KAP theoretical model to assess the public’s knowledge of the correlation between *H. pylori *and gastric cancer. The study found that residents generally had a low level of knowledge about *H. pylori*, with only 24.6% having heard of *H. pylori *and 61% of the participants not knowing the connection between *H. pylori* and gastric cancer. Therefore, the scholar proposed the need to design a new education program to prevent, detect, and intervene in gastric cancer at an early stage by raising public awareness. Regarding the secondary and tertiary prevention stages of gastric cancer, Yang et al. [18] conducted a study on patients after gastric cancer resection based on the KAP theoretical model. The results confirmed that there is a significant positive relationship between knowledge and attitude, knowledge and practice, and attitude and practice (correlation coefficients were found to be r = 0.405, 0.511, and 0.458, respectively; all P < 0.001), indicating that higher knowledge levels, positive attitudes, and longer practice times positively impact preventing the recurrence of gastric cancer.

Challenges of the KAP Theoretical Model in Gastric Cancer Prevention

Difficulty in popularizing knowledge: In the process of promoting knowledge on gastric cancer prevention, we found that some members of the public lacked in-depth understanding and knowledge of this knowledge, resulting in limited prevention effects. For example, in a survey on the correlation between *H. pylori *and gastric cancer, as many as 61% of participants did not know the link between *H. pylori *and gastric cancer [13]. In a study of 1,509 people aged 40-70, it was found that personal background factors such as sociodemographic factors (b = 0.338), economic status (b = -0.127), perceived risk ability (b = 0.048), and past experience (b = 0.103) would affect cognition and lead to difficulties in popularizing knowledge [8].

Resistance to attitude change: Even if the public has acquired certain knowledge about gastric cancer prevention, they are often unable to immediately change their unhealthy lifestyle and dietary practices due to the influence of long-standing lifestyle practices and cultural concepts. A study found that unhealthy lifestyle practices were equally common in cancer patients and non-cancer patients, but there was a difference in the willingness of the two groups to change their unhealthy lifestyle practices (P < 0.05) [9].

Challenges in behavioral practice: Transforming knowledge into actual practice is a key link in the KAP theoretical model. However, multiple factors can affect the occurrence of patient practice. Studies have found that education level, occupation, place of residence, and degree of family support are significantly related to disease-related behavior (P < 0.05), among which the patient’s place of residence and family support are independent influencing factors (P < 0.05) [19].

Countermeasures of the KAP Theoretical Model in the Prevention of Gastric Cancer

Strengthen the targeted dissemination of gastric cancer knowledge: Through in-depth research, we can understand the public’s needs and current knowledge of gastric cancer prevention knowledge and develop more practical and targeted publicity content and strategies. In a report investigating the public’s knowledge of the correlation between *H. pylori *and gastric cancer, it was pointed out that only 45% of the participants believed that bacterial infection may lead to the development of gastric ulcers, 27.8% of the people recognized the connection between the use of non-steroidal anti-inflammatory drugs and the development of gastric ulcers, and about 40% of the people did not recognize the connection between bad practices and lifestyle choices and the development of gastric ulcers. Even 3% of the participants believed that stress may be related to the development of gastric ulcers [7]. New health education activities based on the above survey results will be more targeted and improve the publicity effect. Another report conducted a meta-analysis of 12 included articles and concluded that the content of health education for gastric cancer should include cancer epidemiology, cancer risk factors, warning symptoms, and screening methods [10].

Guide the public to form correct health concepts: Emphasize the harm of bad living and eating practices to health through health education, and introduce scientific and reasonable healthy living practices. Zhao et al. [20] found that the scores of quality of life, physical function, role function, emotional function, cognitive function, and social function of the health education group were higher than those of the control group (P < 0.001; OR (95% CI)). The values are 9.78 (8.59-10.99), 8.13 (6.65-9.60), 8.99 (7.84-10.14), 9.84 (8.64-11.50), 6.55 (5.59-7.71), and 9.79 (8.59-10.99), indicating that health education can significantly improve the quality of life of gastric cancer patients.

Establish a long-term practice promotion mechanism: Provide continuous support and incentives to the public by setting up health clubs and conducting health challenge activities. At the same time, strengthen the supervision role of the community and family, form a multi-party joint force, and jointly promote the public to form a healthy life practice. In addition, scientific and technological means, such as smart health monitoring equipment and health management apps, can be used to provide the public with personalized health management and services for the prevention of gastric cancer [11].

Prospects of the KAP Theoretical Model in Gastric Cancer Prevention

With the development of big data and artificial intelligence technology, the future KAP theoretical model will pay more attention to personalized health education. By analyzing individual health conditions, life practices, psychological characteristics, and other factors, a gastric cancer prevention plan can be tailored for each person to improve the pertinence and effectiveness of health education. Secondly, gastric cancer prevention involves multiple disciplines, such as medicine, nutrition, and psychology. The future KAP theoretical model will pay more attention to interdisciplinary cooperation, integrate resources from all parties to form a joint force, and jointly promote the in-depth development of gastric cancer prevention. Finally, in addition to traditional publicity and education methods, the future KAP theoretical model will explore more diversified intervention methods, such as social media, mobile applications, virtual reality, etc. At present, some scholars have combined ChatGPT with the KAP theoretical model to develop a tool to evaluate the knowledge, attitudes, and practices of ChatGPT in pharmacy practice and education. The tool showed content validity index >0.78, face validity index >0.8, internal consistency reliability Cronbach’s alpha >0.8, and construct validity factor loadings >0.5 [11]. In terms of the KAP theoretical model, we look forward to more solutions that combine artificial intelligence technology to help prevent gastric cancer, so as to better meet the public’s demand for gastric cancer prevention information.

## Conclusions

This article reviews the current application status of the KAP theoretical model in gastric cancer prevention, analyzes the challenges and countermeasures, and looks forward to future development prospects. The KAP theoretical model has important value in gastric cancer prevention by improving health knowledge, changing health attitudes or beliefs, and guiding health practices. At present, the application of this model in gastric cancer prevention has gradually received attention, mainly reflected in health education. However, there are still challenges in knowledge popularization, attitude change, and behavioral practice. To meet these challenges, it is necessary to strengthen the pertinence and effectiveness of gastric cancer knowledge popularization, guide the public to form correct health concepts, and establish a long-term behavior promotion mechanism. In the future, with the development of big data and artificial intelligence technology, the KAP theoretical model will pay more attention to personalized health education, interdisciplinary cooperation, and diversified intervention methods.
